# Effects of imipramine on cancer patients over-expressing Fascin1; description of the HITCLIF clinical trial

**DOI:** 10.3389/fonc.2023.1238464

**Published:** 2023-09-29

**Authors:** Antonio Asensi-Cantó, Edith Rodríguez-Braun, Asunción Beltrán-Videla, Ana María Hurtado, Pablo Conesa-Zamora

**Affiliations:** ^1^Facultad de Ciencias de la Salud, Universidad Católica de Murcia (UCAM), Guadalupe, Spain; ^2^Pharmacy Department, Hospital Universitario Santa Lucía, Cartagena, Spain; ^3^Molecular Pathology and Pharmacogenetics Research Group, Instituto Murciano de Investigación Biosanitaria (IMIB), Hospital Universitario Santa Lucía, Cartagena, Spain; ^4^Oncology Department, Hospital Universitario Santa Lucía, Cartagena, Spain; ^5^Innmunobiology for Aquaculture Research Group, Cellular Biology and Histology Department, Universidad de Murcia, Murcia, Spain; ^6^Laboratory Medicine Department, Hospital Universitario Santa Lucía, Cartagena, Spain

**Keywords:** cancer, metastasis, colorectal cancer, breast cancer, imipramine, Fascin1, tumor invasion, histology

## Abstract

**Background:**

Tumor invasion and metastasis are responsible for the majority of cancer-related deaths. The identification of molecules involved in these processes is crucial to design effective treatments that can halt the progression of cancer. To spread and metastasize, tumor cells must restructure their cytoskeleton and emit protrusions. A key molecule in this process of creating these invading structures is Fascin1, the main protein involved in the formation of actin cytoskeleton bundles and a consistent marker of bad prognosis in several types of cancer. Recent studies have shown that imipramine, an FDA- and EMA-approved antidepressant, can block Fascin1and prevent the formation of actin bundles, making it a promising candidate for the treatment of Fascin1-expressing cancers. As a result, a clinical trial will be conducted to assess the efficacy of imipramine being the first experimental clinical study selecting patients based on Fascin1 expression.

**Methods:**

The HITCLIF trial is a multicenter, double-blind, placebo-controlled, randomized and non-commercial phase II clinical trial conducted in parallel groups to evaluate the effectiveness of the tricyclic antidepressant imipramine as anti-invasive agent in the treatment of localized colon, rectal and triple negative breast cancer patients with overexpression of Fascin1. Eligible patients will be randomly assigned, in a 1:1 ratio, to receive imipramine or placebo. Patients will be stratified into 2 groups according to whether administration of imipramine is concomitant with neoadjuvant chemotherapy regimen. Group A will receive imipramine alone without neoadjuvant chemotherapy, while Group B will receive imipramine treatment along with the standard neoadjuvant chemotherapy regimen. The primary endpoint of the trial is the grade of alteration in the prognostic histopathological features at invasive margins (tumor budding, cytoplasmic pseudo-fragments, tumor growth pattern, and peritumoral lymphocytic infiltration).

**Discussion:**

Fascin1 is an interesting therapeutical target as it plays a causative role in the invasion and metastasis of cancer cells. Moreover, its expression is virtually absent in normal epithelia but highly expressed in cancer with bad prognosis. In silico, *in vitro* and *in vivo* studies by our group have demonstrated that the antidepressant imipramine has Fascin1-dependant anti-invasive and anti-metastatic effects in colorectal cancer cells. Now we are recruiting patients in a clinical trial based on Fascin1 over-expression in which administration of imipramine will be carried out during the period between the diagnosis biopsy and surgical resection to explore the drug effects on tumor invasive front.

**Clinical trial registration:**

https:///www.clinicaltrialsregister.eu/ctr-search/trial/2021-001328-17/ES, identifier 2021-001328-17.

## 1 Introduction

Traditional antineoplastic agents have been designed to attack the most typical characteristic of cancer; i.e., cell proliferation. However, the plasticity of tumor cells and their ability to overcome various obstacles (such as the extracellular matrix, nutrient scarcity, and/or the immune system) pose current challenges to antitumor therapy. For this reason, molecules involved in these tumor adaptation processes and cancer progression are the targets of numerous specific therapies.

In this regard, metastasis, despite being the leading cause of human cancer death, remains elusive to much of the antitumor arsenal currently available (1). This is mainly due to the complexity of the metastatic phenomenon, which involves two phenotypic changes: the epithelial-mesenchymal transition (EMT) and the mesenchymal-epithelial transition (MET). In these processes, the tumor cell does not necessarily proliferate but also migrate and invades. Invasion is a complex event that involves cellular mobility or migration and degradation of the extracellular matrix. In this process, rearrangement of the actin cytoskeleton is crucial for the tumor cell to acquire invasive properties (2).

Fascin1 (encoded by *FSCN1* gene) is a key protein involved in the creation of actin bundles necessary for the construction of invasive cytoskeletal structures and, for this reason, plays a causal role in tumor invasion and metastasis. In fact, epithelial cell tumors (carcinomas and adenocarcinomas) that overexpress Fascin1 are characterized by their greater tendency to metastasize and worse prognosis, while normal epithelial cells barely express this protein (3, 4). Among the tumors in which FSCN1 overexpression has been best characterized are serrated subtype colorectal cancer (5) and triple-negative breast cancer (6).

In addition to their poor prognosis, these two types of tumors are characterized by being refractory to existing molecularly targeted therapies for colorectal cancer (7, 8) and breast cancer, such as anti-EGFR inhibitors, immune checkpoint inhibitors, hormonal therapy, and anti-HER2 drugs. It is therefore necessary to have specific treatments to treat these two types of tumors.

Previous studies have shown that imipramine, a tricyclic antidepressant that inhibits the reuptake of norepinephrine and serotonin approved by the FDA and EMA, is capable of binding to Fascin1, preventing the formation of actin bundles and invasive cellular structures, and reducing invasion and metastasis *in vitro* and in preclinical models (9).

More recently, it has been demonstrated that imipramine exerts an inhibitory effect on tumor growth compared to placebo in a mouse primary tumor model. Previous studies have shown that this antidepressant agent is capable of inhibiting cytoplasmic projections in animal cells through its action against Fascin1, leading researchers to postulate an anti-invasive and anti-metastatic effect for imipramine (10). In addition, there are some reported individual cases of unexpected cures in cancer patients due to imipramine treatment (11), and a study with a cohort of 42,075 patients demonstrating the good adherence to antidepressants and the decrease in premature mortality in cancer patients, including colon and breast cancer (12). However, no biomarkers have been associated to response to imipramine in these studies.

Considering these facts, HITCLIF clinical trial postulates the hypothesis that the inhibition of Fascin1 with imipramine (compared to placebo) could impede or minimize the dissemination of tumor cells from the primary tumor through the stroma, as can be evidenced by assessing histological features of epithelial-mesenchymal transition such as tumor budding and invasive tumor growth pattern.

Imipramine is currently indicated for the treatment of depression, anxiety crises, and chronic pain in adult individuals. Clinical evidence suggests that using imipramine to treat depression or pain in cancer patients is a safe and effective treatment (13–16). Despite the use of antidepressants, including imipramine, in cancer patients, conclusive evidence of their antitumor effects is lacking, possibly due to a lack of patient selection based on a marker like Fascin1. However, several studies conducted in cell lines and animal models have provided a molecular rationale for the potential anti-tumor properties of imipramine (17–20).

Given that overexpression of FSCN1 has been associated with chemotherapy resistance (21) and other Fascin1 inhibitors have proven synergistic effect with chemo- and immunotherapy (22) it is reasonable to believe that inhibition of Fascin1 with imipramine may have these synergistic effects, especially in patients, in the clinical trial context, who will receive neoadjuvant treatment concomitantly with imipramine.

## 2 Materials and methods

### 2.1 Study design

The HITCLIF trial is a multicenter, double-blind, placebo-controlled, randomized and non-commercial phase II clinical trial conducted in parallel group to evaluate the effectiveness of the tricyclic antidepressant imipramine as anti-metastatic agent in the treatment of colon cancer, locally advanced rectal cancer (LARC) and triple negative breast cancer (TNBC) patients with overexpression of Fascin1. The study was initiated by investigators based at the Santa Lucía General University Hospital in collaboration with Biomedical Research Institute of Murcia (IMIB) which is responsible for overseeing the conduct of the trial. The project will be developed in a second public hospital (Virgen de la Arrixaca Clinical University Hospital) also responsible for providing oncological treatments to Murcia Region (Spain). Recruitment started in September 2021 and is estimated to continue until December 2024.

### 2.2 Selection of subjects

#### 2.2.1 Eligibility criteria

The HITCLIF trial is currently recruiting patients with colorectal cancer or TNBC who overexpress Fascin1 in the diagnostic biopsy tissue according to the following criteria.

##### 2.2.1.1 Inclusion criteria

1. Patients aged >18 years willing to participate in the study and provide written informed consent.
2. Newly diagnosed patients with colon cancer (stage II with high-risk features or stage III) and candidates for adjuvant therapy
3. Newly diagnosed patients with LARC or TNBC candidates for neoadjuvant therapy.
4. Overexpression of Fascin1 in primary tumor with immunohistochemistry confirmed.
5. Diagnosis of tumor confirmed by biopsy.
6. Candidates for tumor resection.

##### 2.2.1.2 Exclusion criteria

1. Metastatic disease at diagnosis.
2. Cancer colon with intestinal obstruction or subobstruction.
3. ECOG Performance Status ≥ 3.
4. Inadequate liver function (defined as serum total bilirubin level ≥2 mg/dl) or renal function (defined as glomerular filtration rate < 30 mL/min/1.73m2)
5. History of cardiac disease such as uncontrolled or symptomatic arrhythmias, congenital long QT syndrome or corrected QT interval (QTc) > 480 msec at screening.
6. Comedication with selective serotonin or norepinephrine reuptake inhibitors within 14 days prior to recruitment.
7. Depressive disorder, bipolar depression or psychosis.
8. Pregnant or lactating women.
9. Other conditions that investigators consider not suitable for this study.

### 2.3 Study procedure

**Figure 1:**
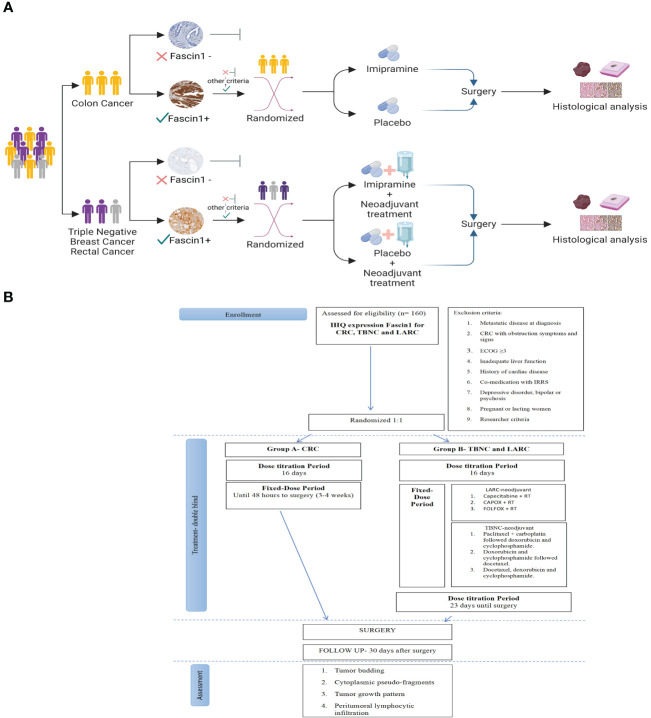
**(A)** Diagram showing the patient selection process for the clinical trial. Created with BioRender.com. **(B)** Consort flow chart that depicts the research clinical trial process.

The HITCLIF trial will involve a two-stage consent process for potential participants (**Figure 1**). In the first stage, patients will be requested to provide their consent for the examination of their resection sample, obtained during biopsy diagnosis. The resection samples will be subjected to FSCN1 immunostaining analysis. Patients who test positive for immunohistochemistry and do not show evidence of metastatic disease will be considered suitable for participation in the HITCLIF trial. Prior to obtaining their consent, the patients will receive a thorough explanation of the trial.

Randomization will take place once the consented patient has completed all the necessary baseline procedures and is deemed eligible for study entry. Treatment assignment will be done using a web-based central randomization system. Eligible patients will be randomly assigned, in a 1:1 ratio, to receive imipramine or placebo. Patients will be stratified into 2 groups according to whether administration of imipramine is concomitant with neoadjuvant chemotherapy regimen:

**Group A** consists of colon cancer patients who will receive imipramine alone without neoadjuvant chemotherapy between their diagnostic biopsy and up to 48 hours before surgical resection. Adjuvant chemotherapy is recommended for patients with stage III disease and is also an option for some patients with high-risk stage II disease. However, patients with specific circumstances like bulky nodal disease or resectable T4b tumors, neoadjuvant therapy may be recommended, but in order to establish a homogeneous group these particular patients will be excluded from the participation in this study. Consequently, Group A patients receive imipramine prior to surgery as monotherapy, without concomitant treatment. The expected treatment duration for Group A is approximately 3 to 4 weeks, depending on the timing between the diagnosis and the intervention appointment date (23).

**Group B** comprises patients with TNBC and LARC who will receive imipramine treatment along with the standard neoadjuvant chemotherapy regimen between their diagnostic biopsy and up to 48 hours before surgical resection. The expected duration of treatment for Group B is dependent on the specific neoadjuvant chemotherapy regimen chosen by the physician and is estimated to be 4 to 6 months.

Regardless of the treatment group, all patients in the study will undergo surgical resection. As previously mentioned, the administration of the study medication should be ceased at least 48 hours prior to the scheduled surgery. All necessary procedures to prepare for surgical intervention, will be conducted in accordance with standard clinical practice.

Following the surgical resection, the tumor resection specimens procured during the intervention will undergo histopathological evaluation to assess the prognostic pathological features (incidence of tumor budding, cytoplasmic pseudo-fragments, tumor growth pattern and peritumoral lymphocytic infiltration). The results of the histopathological assessment are crucial in determining the patient’s prognosis and the research team will record the results into the study electronic case report forms (eCRFs). Access to the eCRFs will be limited to clinical trial staff to ensure patient data confidentiality and security.

### 2.4 Study treatment

Patients will receive continuous oral imipramine 195 mg daily in capsule formulation from randomization until 48 hours before surgical resection. To ensure acceptable tolerability and reduce the likelihood of anticholinergic adverse events, the imipramine dose will be gradually increased from 25 mg to 195 mg daily during the first weeks (**Table 1**). Patients in the control arm will receive an oral placebo capsule according to the same schedule and identical to the intervention. The study drug and placebo will be manufactured, labelled and distributed by the Pharmacy Department of Santa Lucía General University Hospital.

**Table 1:** Dose Escalation Levels in HITCLIF trial.

Dose Levels	1	2	3	4	5
Dose (mg)	25	25	25/65	65	65
Schedule	QD	BID	BID	BID	TID
Total Daily Dose (mg)	25	50	90	130	195
% Change from Previous Dose Level	–	100%	80%	44%	33%

QD, once daily; BID, twice daily; TID, three times a day. colon cancer patients 2 days in each dose level; LARC and TNBC 4 days in each dose level.

The treatment duration for the two groups in the study, A and B, depends on several factors. For Group A, the treatment duration is expected to be approximately 3 to 4 weeks, depending on the timing of the intervention. This is because these patients will receive imipramine alone, without neoadjuvant chemotherapy, between their diagnostic biopsy and up to 48 hours before surgical resection. After surgery, adjuvant chemotherapy, which is the standard of care for colon cancer patients, may be given, but this is not part of the study protocol.

For Group B, the treatment duration is dependent on the specific neoadjuvant chemotherapy regimen chosen by the physician and is estimated to be 4 to 6 months. This is because these patients with TNBC and LARC will receive imipramine treatment along with the standard neoadjuvant chemotherapy regimen between their diagnostic biopsy and up to 48 hours before surgical resection. The duration of the neoadjuvant chemotherapy regimen will be variable based on the type and stage of cancer, as well as the individual patient’s response to treatment. Therefore, the treatment duration for Group B is expected to be longer than that for Group A.

In order to maximize the treatment in Group A and to ensure that the maximum therapeutic benefit is achieved within the limited treatment window available (3-4 weeks), colon cancer patients will be allowed to escalate the dose earlier than patients in Group B. After the dose adjustment, all patients will receive a full dose of 65 mg of imipramine orally three times a day or the maximum tolerated dose as determined by their physicians.

Due to the increased risk of withdrawal symptoms if the antidepressant is stopped suddenly after regular administration for 8 weeks or more, a sequential dose reduction over a 3-week period has been developed to minimize this potential risk in patients with LARC and TNBC. This gradual decrease in dosage during the final 3 weeks of therapy will decrease the likelihood of experiencing withdrawal symptoms. The doses will be reduced to 130 mg, 90 mg, 50 mg, and 25 mg per day, allowing the antidepressant to be safely discontinued.

Furthermore, the study includes a dose de-escalation process in response to the occurrence of adverse events during treatment that is monitored by the clinical trial investigators to ensure patient safety. Patients who experience intolerable side effects or adverse reactions related to the study drug will have their dose descaled in a gradual and sequential process. The investigational drug, imipramine, will be initiated at a dose of 25 mg daily and will be gradually increased up to a maximum of 195 mg daily. If a patient experiences intolerable side effects, the dose will be reduced to 130 mg, 90 mg, 50 mg, and 25 mg daily, based on the patient’s drug tolerance. If the patient is unable to tolerate the minimum concentration, they will be withdrawn from the clinical trial.

In this clinical trial, the Pharmacy Department of Santa Lucía General University Hospital will prepare clinically indiscernible capsules of imipramine and placebo, and distribute the study medication to investigating pharmacies located in participating hospitals. The pharmacies will then dispense the medication to eligible patients under the supervision of the principal investigator. This distribution process is closely monitored to ensure that each patient receives the correct medication and dosage, and to maintain the integrity of the clinical trial results. The experimental drug will be stored in a secure and locked area of the investigating pharmacies, following the specific storage conditions indicated on the product label, to prevent unauthorized access.

### 2.5 Concomitant treatment

In the HITCLIF trial, patients with LARC and TNBC who are eligible for neoadjuvant chemotherapy will receive the standard of care chemotherapy regimen according to clinical guidelines. The chemotherapy will be administered before surgery, and patients will continue to receive the investigational drug, imipramine, alongside the chemotherapy. The neoadjuvant treatment regimen and schedule of hospital visits during chemotherapy will be determined by the physician and will not be influenced by the clinical trial protocol. The concomitant treatments in Group B involves different schedules of preoperative therapy consider standards of care in clinical practice (**Table 2**).

**Table 2:** Concomitant treatments in HITCLIF trial: Preoperative Therapy for Group B.

NEOADJUVANT THERAPHIES FOR RECTAL CANCER	DESCRIPTION OF CHEMORADIOTHERAPY TREATMENT
Long-course radiotherapy with concomitant capecitabine	Radiotherapy in 28 daily fractions of 1,8 Gy up to 50,4 Gy or 25 fractions of 2,0 Gy up to 50,0 Gy, along with concomitant twice-daily oral capecitabine 825 mg/m².
Short-course radiotherapy with CAPOX chemotherapy	Short-course radiotherapy in 5 fractions of 5 Gy over a maximum of 8 days, followed by six cycles of CAPOX chemotherapy. The chemotherapy regimen includes capecitabine 1000 mg/m2 orally twice daily on days 1–14, oxaliplatin 130 mg/m2 intravenously on day 1, and a chemotherapy-free interval between days 15–21.
Short-course radiotherapy with FOLFOX4 chemotherapy	Short-course radiotherapy in 5 fractions of 5 Gy over a maximum of 8 days, followed by nine cycles of FOLFOX4 chemotherapy. The chemotherapy regimen includes oxaliplatin 85 mg/m2 intravenously on day 1, leucovorin (folinic acid) 200 mg/m2 intravenously on days 1 and 2, followed by bolus fluorouracil 400 mg/m2 intravenously and fluorouracil 600 mg/m2 intravenously for 22h on days 1 and 2, and a chemotherapy-free interval between days 3–14.
NEOADJUVANT THERAPIES FOR TRIPLE NEGATIVE BREAST CANCER	DESCRIPTION OF CHEMOTHERAPY TREATMENT
Paclitaxel and carboplatin followed by doxorubicin and cyclophosphamide	Paclitaxel weekly at 80mg/m^2^ for 12 weeks, plus carboplatin (AUC 5/6) on day 1 every 21 days for 4 consecutive cycles, followed by dense-dose doxorubicin at 60-75mg/m^2^ and cyclophosphamide at 600mg/m^2^ on day 1 every 14 days for 4 consecutive cycles to start on week 13.
Doxorubicin and cyclophosphamide followed by docetaxel	Doxorubicin 60 mg/m^2^ plus cyclophosphamide 600 mg/m^2^ every 21 days for 4 consecutive cycles followed by docetaxel 100 mg/m^2^ every 21 days for 4 consecutive cycles to start 21 days after final cycle of doxorubicin and cyclophosphamide.
Docetaxel, doxorubicin and cyclophosphamide	Docetaxel at 75mg/m^2^, doxorubicin at 50mg/m2, and cyclophosphamide at 500mg/m2 on day 1 every 21 days for 6 consecutive cycles.

### 2.6 Study interventions

The schedule of enrolment, interventions, and assessments in accordance with the SPIRIT 2013 statement is shown in **Table 3**.

**Table 3:** Schedule of enrolment, interventions, and assessments according to SPIRIT 2013 statement of defining standard protocol items for clinical trials.

	Enrolment	Allocation	Doble -Blind Period												Surgery		Close-out
	Screening	Day 1	Dose titration Period			Fixed-Dose Period					Dose titration Period				Prior surgery	Surgery	Post-operative day 30
Weeks	Preselection visit	Randomization visit	1	2	3	4	8	12	16	20	21	22	23	24			Follow-up
Time point			Treatment			Treatment					Treatment						
**Procedures**																	
Informed consent Fascin1	**x**																
Inclusion/exclusion criteria	**x**																
Demographic date	**x**																
Medical history	**x**																
Informed consent clinical trail		**x**															
Allocation		**x**															
Dispensing of investigational drug		**x**															
Biomarkers		**x**													**x**		**x**
Group A- CRC			**x**	**x**	**x**	**x**											
Group B-- TBNC/ LARC			**x**	**x**	**x**	**x**	**x**	**x**	**x**	**x**	**x**	**x**	**x**	**x**			
Surgery																**x**	
Return de investigational drug															**x**		
**Assessments**																	**x**
Tumor budding																	**x**
Cytoplasmic pseudofragments (CyPs)																	**x**
Tumorgrowth pattern																	**x**
Lymphocytic infiltrate																	**x**
NGS analysis of the resection tumor																	**x**
cfDNA		**x**													**x**		**x**
Fascin 1 levels in serum		**x**													**x**		**x**

At **Preselection visit**, the complete medical and surgical history of the patient is recorded. The patient will be properly and fully informed and invited to sign the informed consent to FSCN expression analysis of their resection sample. The availability of a tumor specimens collected at diagnosis by biopsy is mandatorily required for participation in this study. The sample will be sent to the Department of Pathology, located at Santa Lucía General University Hospital to proceed with immunohistochemistry analysis of the resection sample.

**Figure 2:**
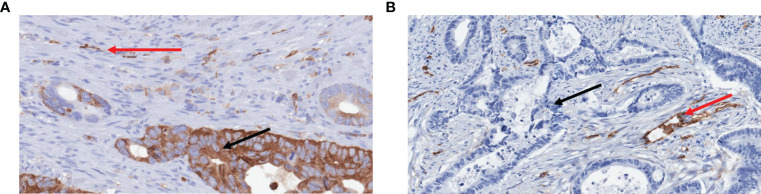
Examples of immunohistochemical expression of Fascin1 in colorectal cancer. **(A)** Positive case showing high intensity and >75% staining distribution (DD score) for Fascin1 in tumour cells (black arrows). **(B)** Negative case showing no Fascin1 expression (AA score) in tumor cells. Note in both examples the internal positive controls represented by endothelial cells and some immune cells (red arrows).

Fascin1 expression will be assessed by immunohistochemistry as described previously (5, 24). Briefly, 1:50 diluted anti-Fascin1 antibody will be purchased from Dako (clone: 55K-2; ref: M356701-8) and immunohistochemistry will be carried out in a BenchMark Ventana autostainer with citrate buffer, pH 6.1, 95°C and 20 min for antigen retrieval procedure. Diffuse cytoplasmic staining in epithelial tumor cells will be considered a positive reaction. Immunohistochemical expression of Fascin1 in vascular endothelium will be considered an internal positive control whereas the absence of expression in normal mucosa as a negative (**Figure 2**).

Staining scores will be calculated by multiplying the staining intensity score (0 = no staining, 1 = weak or moderate, 2 = strong) in a given tumoral area by the stained area score (1 < one-third, 2 ¼ between one- and two-thirds, 3> two-thirds), as previously reported (25). The total immunohistochemical score (0–6) will be expressed as the product of the intensity and area scores. Staining will be considered positive when the score is ≥ 1. Furthermore, a tissue microarray comprising examples of 0-3 scores will be constructed and included in all biopsies to be analyzed. Immunohistochemical assessment will be done without knowledge of histological diagnoses.

After a pathological examination is completed, should the analysis show overexpression of FSCN, the patient will be asked if they wish to participate in the main trial. Before consent is obtained, the patient will be given a full explanation of the trial and offered a Patient Information Sheet.

The **Randomization visit** for the HITCLIF trial will take place once the analysis of the resection sample confirms the overexpression of FSCN, and the patient has completed all the inclusion criteria. At this point, the patient will be stratified into Group A or Group B based on their cancer type and whether they will receive neoadjuvant chemotherapy. After stratification, the patient will be randomized into either the imipramine or placebo arm of the study and the study medication will be dispensed at this visit based on their assigned treatment arm. At this visit, the patient will receive detailed instructions on the administration of the medication, including a specific schedule outlining when and how to take the medication. Additionally, a diary will be provided to the patient to allow for the documentation of any adverse events (AEs) experienced during the treatment.Regarding the assessment and reporting of AEs, the HITCLIF trial protocol states that the trial physician will evaluate all suspected cases of AEs reported by the participants, and all AEs will be recorded in the participants’ medical records and eCRFs using the National Cancer Institute Common Terminology Criteria for Adverse Events. The investigator will report any serious adverse events, defined as AEs that are fatal or life-threatening, require hospitalization, or result in persistent or significant disability/incapacity. The investigator will also report non-serious and serious adverse events of special interest attributed to imipramine in accordance with local procedures, statutes, and the European Clinical Trial Directive.

The following clinical trial visit is the **Pre-surgery Visit**, where patients undergo a preoperative evaluation and preparation for anesthesia and surgical intervention, as part of the standard clinical practice. At this visit, the patients will be requested to discontinue the study treatment and bring the empty trial medication packages and any leftover medication to check their compliance.

The **Surgery Visit** is the fourth scheduled visit in the HITCLIF trial, where tumor resection is performed. The type and extent of resection depend on individual cases, considering factors such as the size and location of the tumor, its invasiveness, and potential complications. Following the surgical intervention, the removed tissue will be sent to the Department of Pathology for subsequent histopathological evaluation.

The last visit in the HITCLIF clinical trial is the **Postoperative Visit**. This visit takes place on Day 30 after the surgical intervention and focus on the management of postoperative complications, monitoring of adverse events, and evaluation of the patient’s recovery progress.

In order of monitoring minimal residual disease using circulating tumor DNA (ctDNA) and measuring serum levels of Fascin1 the patient will undergo blood sampling on three occasions (randomization visit, pre-surgery visit, and postoperative visit). Each collection will entail the extraction of a 10 ml tube for complete blood count (CBC) and another 10 ml tube for serum collection. The randomization visit will entail the only venipuncture performed on the patient for the sole purpose of the clinical trial. Routine clinical practice blood collections will be used during the other visits to minimize patient discomfort and complications.

### 2.7 Assessment

The principal objective of this clinical trial is to evaluate the effect of imipramine treatment on the development of histological features associated with EMT during the time interval from the diagnostic biopsy analysis to the surgical resection intervention. The following prognostic histopathological features at invasive margins will be evaluated: (1) **tumor budding**, (2) **cytoplasmic pseudo-fragments**, (3) **tumor growth pattern** and (4) **peritumoral lymphocytic infiltration**.

**Tumor budding** will be evaluated according to the method previously reported by Ueno H et al. (26), where tumor budding foci is defined as an isolated single tumor cell or a cluster composed of up to 4 tumor cells. For **cytoplasmic pseudo-fragments** evaluation, the presence or absence of cytoplasmic fragments will be assessed through immunostaining for cytokeratin, following the criteria proposed by Shinto et al. (27). The highest number of tumor budding and cytoplasmic pseudo-fragments in an ×20 objective lens field (3.2 mm^2^) will be recorded as the score in each case. The same quantification system for both TB and CyPs, as proposed by Shinto et al.11, will be used: counts of 0–9 will be defined as low-grade (LG), while those of ≥10 will be classified as high-grade (HG). HG-TB will be further subdivided into moderate (10–19 foci) and severe (>20 foci).

**Tumor growth pattern** will be classified into two categories, expanding or diffusely infiltrating, based on the criteria suggested by Jass et al. (28). For tumors that exhibit diffuse infiltration, parameters such as the “streaming dissection” of muscularis propria, dissection of mesenteric adipose tissue by small glands or irregular clusters or cords of cells, or perineurial invasion will be considered as indicative. Tumors that do not demonstrate these criteria will be categorized as expanding.

**Peritumoral lymphocyte infiltration** will be assessed based on the criteria established by Klintrup et al. (29). Absence or mild presence of lymphocytic infiltration will be classified as weak, whereas the presence of band-like or cup-like infiltration of lymphocytes will be classified as intense.

Secondary objectives of the study include monitoring minimal residual disease using ctDNA and quantifying serum levels of Fascin1 compared among the intervention (imipramine) and the control group (placebo).

### 2.8 Sample size

The sample size calculation of HITCLIF trial was performed using the OpenEpi program. The estimated total sample size is 180 patients, which will be distributed as follows: 54 for Group A (colon cancer), with 3 patients receiving imipramine versus 3 patients receiving placebo per participating center (n = 3 hospitals) and per year (n = 3 years of recruitment); and 126 for Group B (rectal cancer and TNBC), with 7 patients receiving imipramine plus neoadjuvant therapy versus 7 patients receiving neoadjuvant therapy alone (Group B).

The estimated frequency of tumors overexpressing FSCN1 is as follows: 14.3% of conventional colorectal carcinomas and 88.6% of serrated colorectal carcinomas overexpress FSCN1 (5). Approximately 80% of colorectal carcinomas are of the conventional type and 10% are of the serrated type. As for TNBC, 87.8% overexpress Fascin1 and represent 20% of all breast carcinomas (30).

### 2.9 Statistical analysis

Statistical analysis for the HITCLIF clinical trial involves descriptive and inferential analyses of demographic and baseline characteristics of the study population. Descriptive statistics, including measures of central tendency and dispersion, will be used to summarize continuous variables, while categorical variables will be reported as frequency tables. The baseline characteristics of the three groups of participants will be compared using appropriate statistical tests, such as the Chi-Square test for categorical variables and the t-Student test for continuous variables. Variables will be expressed as mean ± standard error or 95% confidence interval.

The primary endpoint of the trial is the comparison of changes in invasive front associated with the epithelial-mesenchymal transition, measured by tumor budding, cytoplasmic pseudo-fragments, tumor invasion pattern, and immune infiltration characteristics (peritumoral lymphocytes) between the 2 study groups. Chi-Square tests or Fisher’s exact tests will be used to compare qualitative variables, while non-parametric tests such as Mann-Whitney U or parametric tests such as t-Student will be employed for quantitative variables.

In addition to the primary endpoint, the study will evaluate secondary outcomes such as tissue and serum expression of Fascin1 and the quantification of circulating tumor DNA in plasma. The same statistical tests used for the primary endpoint analysis will be applied to compare these variables between the two study groups.

Statistical analyses will be performed with using IBM SPSS version 22. P-value ≤ 0.05 will be considered a significant level in all statistical tests.

## 3 Current status of the trial

The Spanish Agency for Drugs and Medical Products (AEMPS) approved the final version of the protocol in January 2023. Immunohistochemical expression of Fascin1 was performed in 85 candidate patients, including 53 CRC, 26 with rectal cancer, and 6 with TNBC. The positivity rates for Fascin1 were 41.5% for CRC, 27% for rectal cancer, and 100% for TNBC. The first patient was enrolled in March 2023 and, since then, a total number of six patients have been recruited.

Of these six patients, four patients have been diagnosed with TNBC, one with rectal cancer, and one with colon cancer. So far, adverse effects that might be related to imipramine have been described in one patient with TNBC, being grade 1 and consisting of pruritus in the facial region, rest tremor, brief episode of blurred vision, diaphoresis and hyperhidrosis, probable rash, and xerostomia. In the rest of the patients who presented adverse effects, these were related to their neoadjuvant treatment.

## 4 Discussion

The causative role of Fascin1 in tumor invasion and metastasis, its virtually absent expression in epithelial cells and its association with cancer types characterized by lack of standard molecular targeted therapy and bad prognosis have pointed out this protein as an emerging therapeutical target in cancer. This is especially relevant for TNBC which accounts for 10-20% of all cases of breast cancer but shows Fascin1 expression in 87.8% of cases (30).

This tumor is characterized by the low expression of progesterone receptor, estrogen receptor, and human epidermal growth factor receptor 2, thus making TNBC resistant to receptor- and HER-based molecular targeted therapy.

For the past nine years our group have been identifying and characterizing new Fascin1 inhibitors after demonstrating that serrated adenocarcinoma (SAC), a CRC subtype with bad outcome, typically shows a higher Fascin1 expression that the conventional CRC. Similarly, to TNBC, SAC cases are mainly resistant to molecular targeted therapy such as anti-EGFR and immune checkpoint inhibitors as most SACs carry *KRAS* or *BRAF* mutation and are microsatellite stable (7, 8, 31).

Therefore, our group performed virtual drug screening with in silico libraries containing FDA-approved drugs. This analysis suggested that imipramine could be a potential Fascin1 inhibitor. Subsequent *in vitro* and *in vivo* studies confirmed that assumption. One important advantage of drug repurposing as this is case is that the safety profile of the repurposed drug is optimal. In spite of this fact, we considered convenient to include an escalation and de-escalation regime at the beginning and the end of imipramine administration in cancer patients to minimize the central effect of this antidepressant. This escalating dosage has also been carried out in the other clinical trial using imipramine as an antitumoral agent. It consists of an active, not recruiting, single arm, non-randomized clinical trial which has enrolled 17 enrolled patients having triple negative breast carcinoma (NCT03122444), although there is no selection based on the expression of Fascin1.

In addition to its role in invasion and metastasis, Fascin1 expression has been associated also with chemotherapy resistance (21) so Fascin1 inhibitor may potentiate the effect of standard of care therapy (32, 33). In fact, recently, Wang et al. have reported the first documented synergistic effect between the Fascin1 inhibitor NP-G2-044 and the immune checkpoint inhibitor anti-PD1. Their study demonstrates that Fascin1 blockade not only enhances the anti-tumor immune response but also has a reinvigorating effect. This effect was observed in syngeneic mouse models of various cancers including TNBC, lung cancer, pancreatic cancer, prostate cancer, and later in bladder cancer. The study also revealed that conventional dendritic cells (cDCs) in the tumor microenvironment TME express high levels of Fascin1 and the treatment with NP-G2-044 resulted in an increase in intratumoral-activated cDCs and enhanced antigen uptake by these cells (22).

Two clinical trials, NCT03199586 and NCT05023486, have been conducted to evaluate the efficacy of NP-G2-044 as a monotherapy or in combination with anti-PD1 in patients with advanced or metastatic solid tumors. The first trial is a completed phase 1 study, while the second trial is currently in the recruiting phase.

Therefore, it is reasonable to believe that inhibition of Fascin1 with imipramine may have synergistic effects, especially in patients who, in our clinical trial context, will receive neoadjuvant treatment concomitantly with imipramine (i.e., rectal and triple-negative breast cancers).

The reason for evaluating the impact of imipramine in the neoadjuvant setting is to obtain results within a feasible timeframe between diagnosis and surgery. Once the impact of imipramine in the neoadjuvant setting has been addressed in this clinical trial, it will be interesting to evaluate whether imipramine is also valuable in other contexts such as combination with radical chemoradiotherapy, palliative chemotherapy, and maintenance therapy.

The clinical trial presented here could be an interesting starting point to test the use of this antidepressant in other types of tumors in addition to colorectal and breast cancers. Likewise, if the treatment proves good response, it could be applied in the adjuvant context in which clinical endpoints such as disease free- or overall survivals could be obtained to assess the efficacy of imipramine in cancer patients.

## Ethics statement

The studies involving human participants were reviewed and approved by Drug Research Ethics Committee (CEIm) from Hospital Clínico Universitario Virgen de la Arrixaca and from the Spanish Agency of Medicines and Medical Devices (AEMPS). The patients/participants provided their written informed consent to participate in this study.

## Author contributions

AA-C and AB-V are involved in the follow-up of the clinical trial. ER-B and PC-Z wrote the clinical trial protocol. AA-C, AH and PC-Z wrote the manuscript. All authors contributed to the article and approved the submitted version.
