# Erratum: Effects of imipramine on cancer patients over-expressing Fascin1; description of the HITCLIF clinical trial

**DOI:** 10.3389/fonc.2024.1405306

**Published:** 2024-04-16

**Authors:** Frontiers Production Office

**Affiliations:** Frontiers Media SA, Lausanne, Switzerland

**Keywords:** cancer, metastasis, colorectal cancer, breast cancer, imipramine, Fascin1, tumor invasion, histology

Due to a production error, the article type of this article mislabeled as Clinical Trial. The correct article type is Study Protocol.

The publisher apologizes for this mistake.

The original version of this article has been updated.

The publisher apologizes for this mistake. The original version of this article has been updated.

